# Continuous Monitoring of Heart Rate Variability and Respiration for the Remote Diagnosis of Chronic Obstructive Pulmonary Disease: Prospective Observational Study

**DOI:** 10.2196/56226

**Published:** 2024-07-18

**Authors:** Xiaolan Chen, Han Zhang, Zhiwen Li, Shuang Liu, Yuqi Zhou

**Affiliations:** 1 Guangdong Basic Research Center of Excellence for Structure and Fundamental Interactions of Matter, Guangdong Provincial Key Laboratory of Quantum Engineering and Quantum Materials School of Physics South China Normal University Guangzhou China; 2 Guangdong Provincial Engineering Technology Research Center of Cardiovascular Individual Medicine and Big Data, School of Electronic and Information Engineering South China Normal University Foshan China; 3 Key Laboratory of Reproductive Health National Health Commission of the People's Republic of China Institute of Reproductive and Child Health Peking University Beijing China; 4 Department of Pulmonary and Critical Care Medicine Guangdong Provincial Technology Research Center of Chronic Obstructive Pulmonary Disease Rehabilitation Third Affiliated Hospital of Sun Yat-sen University Guangzhou China

**Keywords:** continuous monitoring, chronic obstructive pulmonary disease, COPD diagnosis, prospective study, ROC curve, heart rate variability, respiratory rate, heart rate, noncontact bed sensors

## Abstract

**Background:**

Conventional daytime monitoring in a single day may be influenced by factors such as motion artifacts and emotions, and continuous monitoring of nighttime heart rate variability (HRV) and respiration to assist in chronic obstructive pulmonary disease (COPD) diagnosis has not been reported yet.

**Objective:**

The aim of this study was to explore and compare the effects of continuously monitored HRV, heart rate (HR), and respiration during night sleep on the remote diagnosis of COPD.

**Methods:**

We recruited patients with different severities of COPD and healthy controls between January 2021 and November 2022. Vital signs such as HRV, HR, and respiration were recorded using noncontact bed sensors from 10 PM to 8 AM of the following day, and the recordings of each patient lasted for at least 30 days. We obtained statistical means of HRV, HR, and respiration over time periods of 7, 14, and 30 days by continuous monitoring. Additionally, the effects that the statistical means of HRV, HR, and respiration had on COPD diagnosis were evaluated at different times of recordings.

**Results:**

In this study, 146 individuals were enrolled: 37 patients with COPD in the case group and 109 participants in the control group. The median number of continuous night-sleep monitoring days per person was 56.5 (IQR 32.0-113.0) days. Using the features regarding the statistical means of HRV, HR, and respiration over 1, 7, 14, and 30 days, binary logistic regression classification of COPD yielded an accuracy, Youden index, and area under the receiver operating characteristic curve of 0.958, 0.904, and 0.989, respectively. The classification performance for COPD diagnosis was directionally proportional to the monitoring duration of vital signs at night. The importance of the features for diagnosis was determined by the statistical means of respiration, HRV, and HR, which followed the order of respiration > HRV > HR. Specifically, the statistical means of the duration of respiration rate faster than 21 times/min (RRF), high frequency band power of 0.15-0.40 Hz (HF), and respiration rate (RR) were identified as the top 3 most significant features for classification, corresponding to cutoff values of 0.1 minute, 1316.3 nU, and 16.3 times/min, respectively.

**Conclusions:**

Continuous monitoring of nocturnal vital signs has significant potential for the remote diagnosis of COPD. As the duration of night-sleep monitoring increased from 1 to 30 days, the statistical means of HRV, HR, and respiration showed a better reflection of an individual's health condition compared to monitoring the vital signs in a single day or night, and better was the classification performance for COPD diagnosis. Further, the statistical means of RRF, HF, and RR are crucial features for diagnosing COPD, demonstrating the importance of monitoring HRV and respiration during night sleep.

## Introduction

Chronic obstructive pulmonary disease (COPD) is a prevalent chronic respiratory disease characterized by persistent airflow restriction, with main symptoms including chronic cough, expectoration, and dyspnea that may not be apparent in the early stages of the disease [1]. In recent years, there has been an increasing trend in the prevalence rate of COPD, and large numbers of cases and deaths have been reported. A cross-sectional study conducted in 2007 among 20,245 adults from 7 provinces in China revealed a COPD prevalence rate of 8.2% among individuals older than 40 years [1]. In 2018, data from 2 large epidemiological surveys in China revealed a prevalence rate of ~13.6% among individuals older than 40 years [2,3]. According to the 2019 Global Burden of Disease Study, the global mortality due to COPD reached 3.3 million individuals, with China alone accounting for over 1 million deaths, representing approximately one-third of the total [4]. Due to dyspnea and other symptoms, the daily activities and exercise ability of patients with COPD are greatly limited, which not only seriously affects their quality of life but also imposes a substantial economic burden on the families and society [5,6].

According to the guidelines for the diagnosis and treatment of COPD in China, the current clinical diagnosis methods of COPD primarily include the pulmonary function test, chest imaging examination, pulse oxygen saturation monitoring, arterial blood gas analysis, electrocardiogram, and echocardiography. Among these methods, the pulmonary function test is the gold standard for diagnosing COPD by assessing the degree of airflow limitation and evaluating disease severity. If the ratio of the forced expiratory volume in the first one second to the forced vital capacity of the lungs is less than 70% after inhalation of the bronchodilator, one will be considered to have persistent airflow limitation and can be further diagnosed in conjunction with the clinical symptoms, signs, and history of exposure to risk factors [7].

Patients with COPD commonly exhibit autonomic nervous dysfunction, and the heart rate variability (HRV) serves as a valuable indicator for assessing autonomic nervous function by reflecting the tension and balance between the sympathetic and parasympathetic nerves [8]. Previous studies [9,10] have compared the resting heart rate (HR) and respiratory rate (RR) monitored during daytime between patients with COPD and healthy individuals, and the results suggested that HR and RR can be used as critical indicators for the auxiliary diagnosis of COPD.

In recent years, the rapid development of sensing technology has facilitated the employment of wearable devices for vital signs monitoring [11]. Pioneer studies have demonstrated that the vital signs (including HRV, HR, and respiration) monitored using wearable sensors are identical to those recorded by medical devices [12]. Thus, wearable sensors–aided continuous monitoring is applicable to patients with chronic disease at home [13,14]. Long-term continuous monitoring with wearable sensors during night sleep can effectively record the vital signs of patients [15,16]. Specifically, Bellos et al [17] utilized wearable devices and external devices from the CHRONIOUS platform system to record vital signs such as blood pressure and blood glucose levels and other short-term information of 30 patients with COPD. A mixed classifier was utilized after performing data fusion to classify the severity of COPD among patients, and it yielded an accuracy of 94%. The principal component analysis–based classification using the daytime-recorded vital signs, including HR, respiration, and blood pressure from 8 healthy individuals and 47 patients with COPD, was examined to assess the severity of COPD, and this yielded an accuracy of 88% [18]. In addition to using HR and respiration, Rahman et al [19] considered 30-minute recorded daytime HRV as a feature by using smartwatches to aid in the diagnosis of COPD, yielding a classification accuracy around 80%. By analogy, with daytime-recorded HRV and HR by using wearable devices, Tiwari et al [20] attempted to predict the acute exacerbation of COPD and verified the feasibility of remote sensing–based early warning. The aforementioned studies were based on wearable device–based vital signs recording during daytime in a single day. However, wearable devices have limitations for recording vital signs during night sleep. In addition, studies on diagnosis of COPD based on long-term monitored vital signs (over weeks or even months) have not been reported yet.

Based on the above concerns, we propose to explore the potential of long-term monitored vital signs (including HR, HRV, and respiration) during night sleep to assist in the remote diagnosis of COPD. To the best of our knowledge, this is the first prospective study based on the continuous monitoring of nocturnal vital signs for the remote diagnosis of COPD. Specifically, the HR, HRV, and respiration in patients with COPD and healthy individuals were recorded with bed sensors from 10 PM to 8 AM on the forthcoming day, and the continuous monitoring of each patient lasted no less than 30 days. With the continuously monitored vital signs, the statistical means of HR, HRV, and respiration over the time periods of 7, 14, and 30 days were extracted as features for the diagnosis of COPD.

## Methods

### Study Sites and Participants

A prospective observational study was conducted to recruit case and control individuals from January 2021 to November 2022. The case group comprised of patients with confirmed COPD, while the control group consisted of individuals without any definite diagnosis, and both these groups were recruited from the Guangdong Provincial Technology Research Center of COPD Rehabilitation, the Third Affiliated Hospital of Sun Yat-sen University. Specifically, the case group was derived from the outpatient, emergency, and inpatient departments of this hospital, and the control group consisted of individuals recruited by the Guangdong Provincial Technology Research Center of COPD Rehabilitation. Pulmonary function examination was performed either at or prior to the enrollment of the case group. According to the 2022 Global Initiative for Chronic Obstructive Lung Disease (GOLD) diagnostic criteria, if the ratio of the forced expiratory volume in the first one second to the forced vital capacity of the lungs is less than 70% after inhalation of the bronchodilator, one will be considered to have persistent airflow limitation [21]. This is the gold standard for COPD diagnosis and the only inclusion criterion for case groups. The exclusion criteria for the case and control groups were as follows:

1. Participants younger than 45 years
2. Individuals exhibiting poor compliance, dropouts, or loss to follow-up, including those in coma, in shock, with multiple organ failure, or with a history of epilepsy or any other condition that hinders continued participation in the monitoring study; also excluded were individuals who did not utilize monitoring equipment and those with missing lung function indicators
3. Patients with a history of severe lung diseases such as lung cancer, tuberculosis, severe pulmonary heart disease, and any other malignant tumor disease; individuals with severe cardiovascular diseases, including myocardial infarction, heart failure, arrhythmia, hypertension, and coronary heart disease, and patients with diabetes
4. Individuals with irregular schedules.

### Ethics Approval

This study obtained the written informed consent from all participants, and the study protocol was reviewed by the medical ethics committee of the Third Affiliated Hospital of Sun Yat-sen University (approval [2021]02-019-01).

### Data Collection and Processing

**Figure 1:**
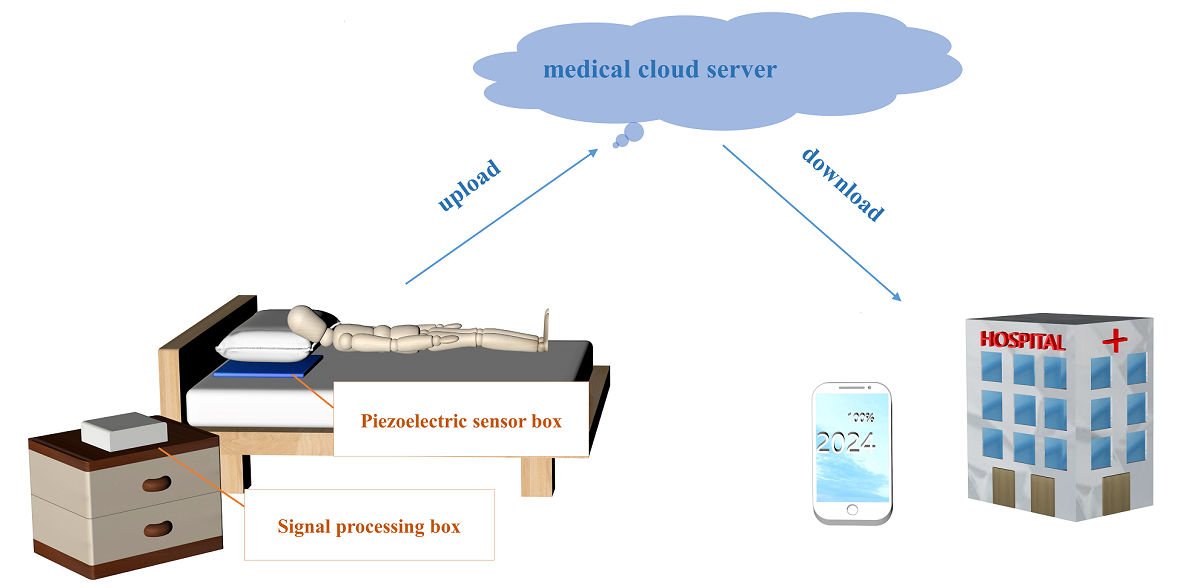
Architecture of the proposed study.

We obtained the pertinent data of individuals in the case group through the hospital information management system, including demographic characteristics, disease history, pulmonary function test data, and acute exacerbations. We also recorded the demographic characteristics and the disease history of participants in the control group. Piezoelectric sensor–based HR and respiratory recorders (model: WSM-LN-03, medical device; China Food and Drug Administration; 20182071130) were distributed to the participants and placed under the pillow to continuously record the HR, respiration, and HRV during night sleep from 10 PM to 8 AM the next day. Prior to the examination, all participants passed the standardized training on how to use the vital sign monitoring device. The recorded vital signs were manually uploaded to the medical cloud server for further data analysis. The architecture of the proposed study is shown in Figure 1. Due to the long duration of the experiment, there may be cases of inevitable data loss. Therefore, specific exclusion criteria with regarding to the data on the medical cloud server were as follows:

1. Number of artifact motions during night sleep ≥600
2. Sleep duration less than 5 hours or longer than 9 hours
3. Data loss in 1 night sleep ≥1 hour
4. Outliers such as HR or RR is 0
5. Data obtained 7 days before and during cases of acute exacerbation of COPD

### Principle of Device and Feature Description

The distributed vital signs monitoring device was deployed under the pillow. With piezoelectric sensors, the analogy signals representing cardiac and respiratory activities are recorded with a sampling frequency of 1 kHz. Specifically designed filter banks can detect the heartbeat interval and respiration per minute during night sleep, and the averaged HR (beat/min) and respiration (times/min) of a whole night can be obtained [22-24]. In addition, by taking advantage of the heartbeat interval, both time and frequency domain HRV parameters can be calculated, as illustrated in Table 1 [25]. Specifically, HRV contains 7 times/frequency domain features, including standard deviation of heartbeat interval (SDNN), total power of heartbeat interval (TP), low frequency of TP (LF), high frequency of TP (HF), low frequency/high frequency (LF/HF), very low frequency of TP (VLF), and ultralow frequency of TP (ULF). The features of respiration also contain the cumulative duration of RR faster than 21 times/min (RRF).

**Table 1:** Feature descriptions of heart rate variability, heart rate, and respiration.

Category, abbreviation of feature		Definition
**Heart rate variability [25]**		
	SDNN	Standard deviation of heartbeat interval
	TP	Total spectral power of heartbeat interval
	LF	Low frequency band power of 0.04-0.15 Hz
	HF	High frequency band power of 0.15-0.40 Hz
	ULF	Ultralow frequency band power of <0.0033 Hz
	VLF	Very low frequency band power of 0.0033-0.04 Hz
	LF/HF	Ratio of low frequency band power over high frequency band power
**Heart rate**		
	HR	Heart rate mode during night sleep (beats/min)
**Respiration**		
	RR	Respiratory rate mode during night sleep (times/min)
	RRF (min)	Cumulative duration of respiratory rate faster than 21 times/min

Since the bed sensors continuously monitor during night, HRV, HR, and respiration can be continuously monitored over weeks without affecting the lifestyle of the recruited patients. Considering that the features of HRV, HR, and respiration obtained in a single night sleep monitoring could be influenced by the daytime emotions and health status of the patients, we propose to use the statistical average of the continuously monitored features (HRV, HR, and respiration) over weeks instead of that recorded in a single day for further analysis. Numerically, the statistical average of the features HRV, HR, and respiration over t consecutive days are calculated by the following method. We first calculated the sum of the values of HRV, HR, and respiration for t consecutive days; then subtracted the maximum and minimum values in the values of HRV, HR, and respiration for t consecutive days; and then obtained the statistical means by dividing by t–2. In this study, we set *t*=1, 7, 14, and 30 days as the cutoff observation time for analysis.

### Sample Size Estimation

The purpose of this study was to evaluate the diagnostic value of continuous HRV and respiration monitoring in patients with COPD and control groups by using pulmonary function examination as the diagnostic gold standard. We expected continuous HRV and respiration monitoring to diagnose COPD with sensitivity of 90%, tolerance error of 10% of sensitivity, specificity of 90%, tolerance error of 10% of specificity, and the test criteria α=.01. The sample size for patients with COPD and control groups was set at 1:2, and the dropout rate was set as 10%. Finally, we used PASS 11.0 software (NCSS Statistical Software) to calculate the sample size, and we found that there should be at least 37 patients with COPD and 74 controls, for a total of 111 participants.

### Statistical Analysis

Statistical analysis was conducted using SPSS software (version 20.0; IBM Corp). The main statistical methods included descriptive analysis, *χ*² test, nonparametric test, binary logistic regression, and receiver operating characteristic (ROC) curves. For statistical description, counting data were described by frequency and rate, and metrological data were described by median and quartile. For statistical tests, the *χ*² test was employed to compare the 2 groups in terms of the counting data, with the *χ*² value serving as the test statistic. The metrological data were analyzed using a nonparametric test, specifically the Mann-Whitney *U* test. The *z* score was employed as the test statistic. In the multifactor analysis, the classical binary logistic regression was applied for classification, and the ROC curve was used to evaluate the diagnosis of each variable. The determination of the cutoff values relied on the maximum Youden index as the tangent point. The test criterion was set as α=.05, and *P*<.05 was considered statistically significant. The classification cutoff point was set as 0.5.

### Data Cleaning and Analysis Flowchart

**Figure 2:**
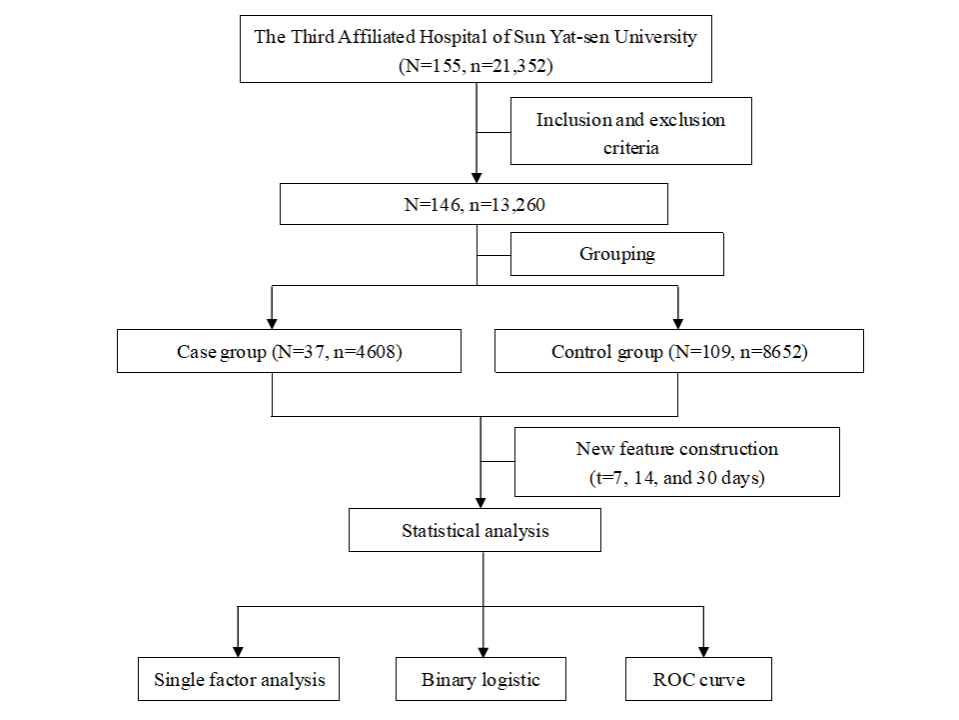
Flowchart of data cleaning and analysis. N: number of participants; n: number of person-times; ROC: receiver operating characteristic; t: cutoff observation time.

The experimental process of this study is shown in Figure 2. We recruited 155 participants corresponding to a total number of 21,352 night-sleep monitored samples (person-time) of patients with COPD and healthy participants in the Third Affiliated Hospital of Sun Yat-sen University, in accordance with the abovementioned inclusion criteria for the population. After data cleaning based on the abovementioned exclusion criteria, 146 patients corresponding to a final number of 13,260 samples (person-time) were included, corresponding to case and control groups of 4608 and 8652 samples, respectively. Subsequently, the statistically averaged features of long-term HRV, HR, and respiration over the observation time of 7, 14, and 30 days were constructed for further analysis.

## Results

### Baseline Characteristics

A total of 146 individuals who met the inclusion and exclusion criteria were enrolled in this study, comprising 37 cases and 109 controls. There were 12 mild to moderate cases and 25 severe cases. The overall compliance rate among the participants was 0.73 (IQR 0.50-0.86), with no statistically significant difference between the 2 groups (*z* score=–1.5; *P*=.15). Moreover, the average number of continuous night-sleep monitoring days per person was found to be 56.5 (IQR 32.0-113.0) days, showing no significant difference between the 2 groups again (*z* score=0.5; *P*=.65). Therefore, compliance and monitoring duration between the 2 groups were comparable. Significant differences were observed between the 2 groups in terms of age, gender, and BMI (*P*<.001). Specifically, the case group exhibited a higher proportion of older individuals, males, and those with low BMI in comparison to the control group (as depicted in Table 2).

**Table 2:** Overall baseline characteristics of the total population.

Variables		Total (N=146)	Control group (n=109)	Case group (n=37)	*z* score/*χ*² *(df)*		*P* value	
Compliance rate, median (IQR)		0.73 (0.50-0.86)	0.76 (0.53-0.86)	0.65 (0.43-0.83)	–1.5		.15	
Monitoring per person, median (IQR)		56.5 (32.0-113.0)	56.0 (34.0-107.0)	78.0 (25.0-164.0)	0.5		.65	
Age (years), median (IQR)		59.0 (52.0-68.0)	55.0 (51.0-63.0)	73.0 (66.0-77.0)	7.0		<.001	
**Sex, n (%)**						21.8 (1)		<.001
	Male	70 (47.9)	40 (36.7)	30 (81.1)				
	Female	76 (52.1)	69 (63.3)	7 (18.9)				
**BMI (kg/m^2^), n (%)**						18.9 (2)		<.001
	<18.5	8 (5.5)	2 (1.8)	6 (16.2)				
	18.5-24	95 (65)	67 (61.5)	28 (75.7)				
	≥24	43 (29.5)	40 (36.7)	3 (8.1)				

### Statistical Comparisons of the Features Between Case and Control Groups

This study consists of 13,260 samples (person-time), corresponding to 8652 in the control group and 4608 in the case group. During the monitoring period of 7, 14, and 30 days, there was a total of 12,396, 11,422, and 9404 person-times recorded, respectively. In 4 cutoffs of the observation time of 1, 7, 14, and 30 days, the distributions of HRV, HR, and respiration features, that is, SDNN, TP, LF, HF, VLF, ULF, HR, RR, and RRF, averaged over different timescales were of significant statistical difference, as shown in Tables S1-S4 of Multimedia Appendix 1. Specifically, the values of all the averaged features in the different timescales in the case group were significantly higher than those of the control group (*P*<.001). Interestingly, no significant difference was observed in the distribution of the features LF/HF between the 2 groups (*P*>.05). Consequently, when performing a binary logistic regression analysis, all features of LF/HF averaged over different timescales (*t*= 1, 7, 14, and 30 days), that is, LF/HF_1_, LF/HF_7_, LF/HF_14_, and LF/HF_30_ were excluded, while the rest 36 features were included.

### Classification Performance of Multidimensional Vital Sign Features

**Figure 3:**
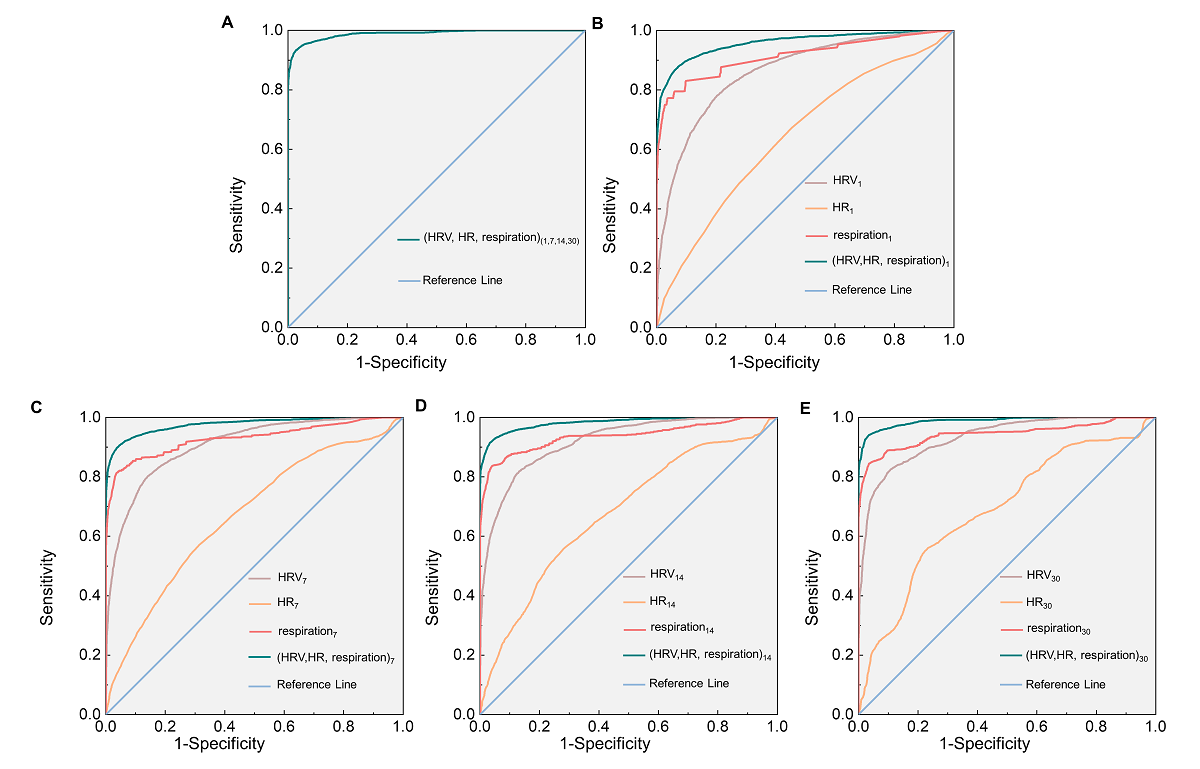
Receiver operating characteristic curve of multidimensional features based on 4 timescales (1, 7, 14, and 30 days; shown in subscripts in the graph). HR: heart rate; HRV: heart rate variability.

HRV, HR, and respiratory features at different timescales are represented by features and timescales, where timescales are in subscripts. The overall classification accuracy of HRV, HR, and respiration at 1, 7, 14, and 30 days achieved a high level of 0.958, with an impressive Youden index of 0.904 and an excellent area under the ROC curve (AUC) of 0.989 (as presented in Table 3 and Figure 3A).

**Figure 4:**
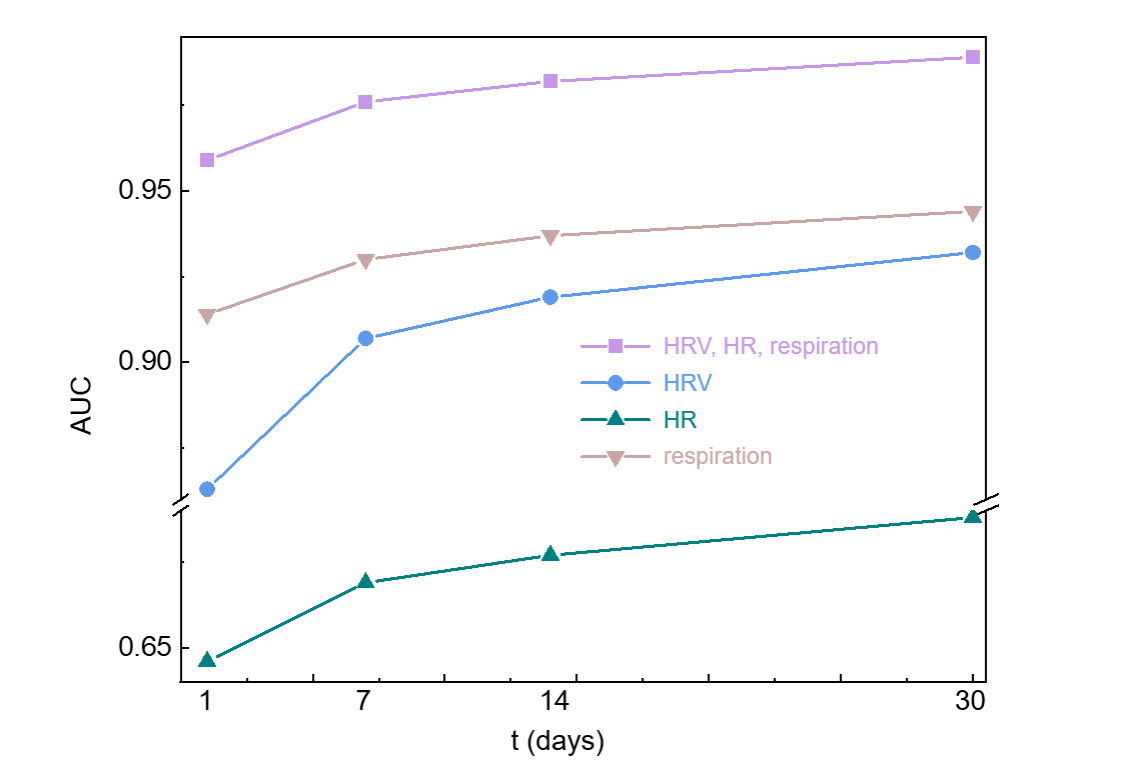
Relationship between area under the curve and monitoring duration of multidimensional statistical features. AUC: area under the curve; HR: heart rate; HRV: heart rate variability; t: time.

We combined the vital signs features based on 4 different timescales (*t*= 1, 7, 14, and 30 days) and subsequently classified the participants into case and control groups. Figure 4 demonstrates that the classification performance of HRV, HR, and respiration improved as the monitoring duration increased, with HRV, HR, and respiration at 30 days exhibiting superior performance (accuracy 0.956, Youden index 0.899, AUC 0.989). Specifically, the ranking of the performance was presented as follows: HRV, HR, and respiration in 30 days > HRV, HR, and respiration in 14 days > HRV, HR, and respiration in 7 days > HRV, HR, and respiration in 1 day.

We combined them again based on the vital signs category and subsequently classified those participants into the case and control groups. Figure 4 demonstrates an improving trend in the classification efficacy of respiration, HRV, and HR, surpassing that of single-day monitoring. On the one hand, when evaluating the classification effect using accuracy and Youden index, we observed an improvement in the classification effect of HRV with the increasing duration of monitoring, where HRV_30_ demonstrated the highest performance (accuracy 0.866, Youden index 0.717). Similarly, as the monitoring duration increased, respiration also exhibited enhanced performance, with respiration at 30 days achieving the best results (accuracy 0.913, Youden index 0.792). However, there was minimal variation in the classification performance of HR across different timescales, maintaining an accuracy rate of approximately 0.67 and a Youden index of around 0.15. On the other hand, AUC was utilized to be evaluated. The findings demonstrated that all AUC values exceeded 0.5 and exhibited statistical significance. Notably, the classification performance of HRV improved with longer monitoring duration, specifically presented as HRV_30_ > HRV_14_ > HRV_7_ > HRV_1_. Similarly, the performance of respiration gradually tended to increase with extended monitoring duration, indicated by respiration over 30 days/respiration over 14 days > respiration over 14 days/respiration over 7 days > respiration over 1 day. However, there were no statistically significant differences between respiration over 14 days and respiration over 7 days or between respiration over 14 days and respiration over 30 days. Furthermore, the performance of HR also progressively enhanced with prolonged monitoring time (HR_30_/HR_14_/HR_7_ > HR_1_). Nevertheless, there were no statistically significant differences among HR_7_, HR_14_, and HR_30_, as shown in Table 3.

Table 3 and Figures 3B-3E demonstrate that the classification performance, as evaluated by the accuracy, Youden index, and AUC, consistently showed respiration > HRV > HR over a single timescale. This consistent pattern was observed over all 4 timescales. In addition, the sensitivity of HR remained consistently low over the 4 timescales, ranging from approximately 0.2 to 0.3, indicating a high potential for missed diagnoses rate (1–sensitivity) of up to 0.7-0.8. Specifically, the sensitivity of HR_1_ was 0.177, with a corresponding missed diagnosis rate of 0.823. These findings highlight that only using HR features for the diagnosis of patients with COPD may lead to a significant increase in missed diagnosis rates exceeding 80%.

**Table 3:** The model evaluation index for the diagnosis of chronic obstructive pulmonary disease by using a combination of multidimensional features.

Features (time=1, 7, 14, 30 days)	Accuracy	Sensitivity	Specificity	Youden index	Area under the curve (95% CI)	*P* value
(HRV^a^, HR^b^, respiration) in 1, 7, 14, and 30 days	0.958	0.924	0.980	0.904	0.989 (0.987-0.991)	<.001
(HRV, HR, respiration) in 1 day	0.915	0.813	0.970	0.783	0.959 (0.955-0.963)	<.001
(HRV, HR, respiration) in 7 days	0.941	0.876	0.976	0.852	0.976 (0.973-0.979)	<.001
(HRV, HR, respiration) in 14 days	0.946	0.892	0.978	0.870	0.982 (0.980-0.985)	<.001
(HRV, HR, respiration) in 30 days	0.956	0.919	0.980	0.899	0.989 (0.987-0.990)	<.001
HRV in 1 day	0.802	0.619	0.899	0.518	0.863 (0.856-0.869)	<.001
HRV in 7 days	0.841	0.732	0.900	0.632	0.907 (0.901-0.912)	<.001
HRV in 14 days	0.851	0.773	0.896	0.669	0.919 (0.914-0.924)	<.001
HRV in 30 days	0.866	0.823	0.894	0.717	0.932 (0.927-0.937)	<.001
HR in 1 day	0.670	0.177	0.932	0.109	0.646 (0.636-0.656)	<.001
HR in 7 days	0.674	0.227	0.920	0.147	0.669 (0.659-0.679)	<.001
HR in 14 days	0.672	0.266	0.905	0.171	0.677 (0.666-0.687)	<.001
HR in 30 days	0.648	0.325	0.858	0.183	0.688 (0.677-0.699)	<.001
Respiration in 1 day	0.889	0.721	0.978	0.699	0.914 (0.908-0.920)	<.001
Respiration in 7 days	0.902	0.767	0.975	0.742	0.930 (0.924-0.935)	<.001
Respiration in 14 days	0.907	0.789	0.975	0.764	0.937 (0.932-0.943)	<.001
Respiration in 30 days	0.913	0.819	0.973	0.792	0.944 (0.939-0.950)	<.001

^a^HRV: heart rate variability.

^b^HR: heart rate.

### Classification Performance of Single Features for 7 Days Long-Term Monitoring

The results shown in Figure 4 indicate that the classification performance using the 7-day monitored features (HRV, HR, respiration; AUC=0.976) is already comparable to that of the 30-day monitored features (HRV, HR, respiration; AUC=0.989). Therefore, considering that the performance of monitoring longer than 7 days tends to saturate, while increasing the monitoring time leads to an increase in the dropout rate, we recommend continuous monitoring for 7 days during daily home life, which can already provide valuable assistance in the auxiliary diagnosis of COPD.

We classified the case and control groups by a single measured vital sign feature during the 7-day long-term monitoring period. As shown in Table 4, the results revealed that the HF_7_ exhibited the most effective classification performance (accuracy 0.830, Youden index 0.602), followed by TP_7_ (accuracy 0.792, Youden index 0.474) among the 6 features (HRV) evaluated by the accuracy and Youden index. Among the features set (HRV, HR, respiration)_7_, RRF_7_ demonstrated the best performance (accuracy 0.898, Youden index 0.733), followed by HF_7_, while HR_7_ showed the poorest performance (accuracy 0.674, Youden index 0.147). Furthermore, both HR_7_ and ULF_7_ exhibited very low sensitivity with the missed diagnosis rate exceeding 70%, indicating that using HR_7_ or ULF_7_ for classification and prediction could lead to higher chances of missed diagnosis.

**Figure 5:**
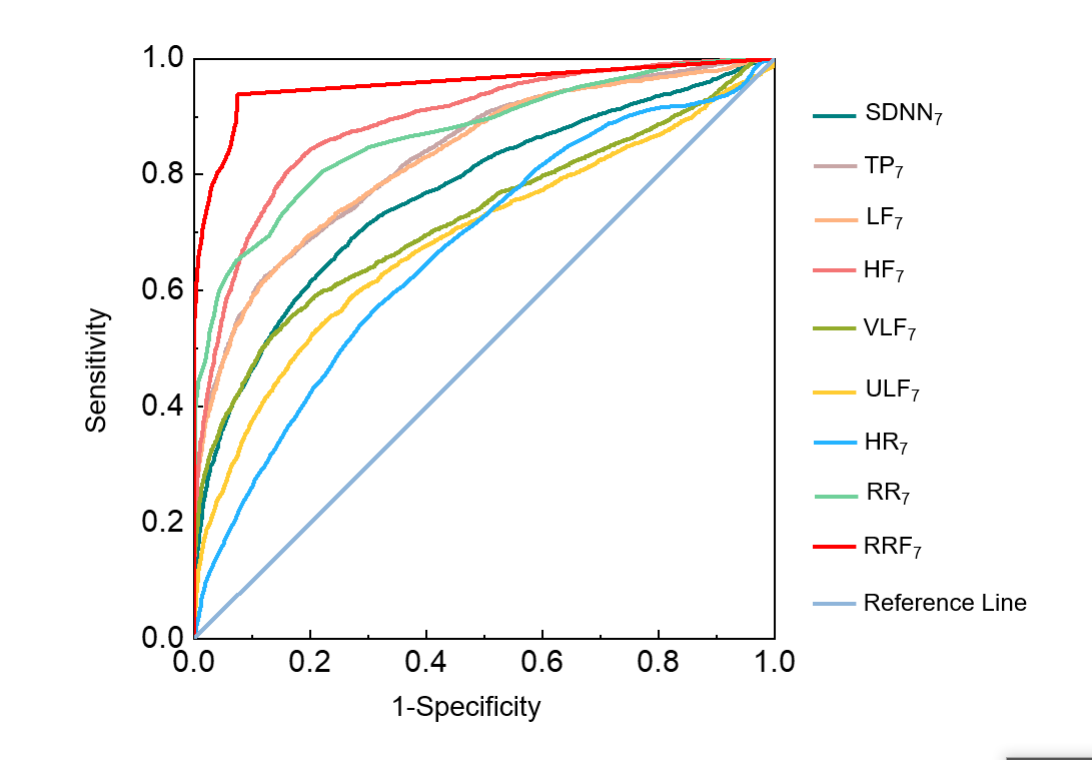
Receiver operating characteristic curve of single statistical average of features based on 7 days monitoring. HF: high frequency band power of 0.15-0.40 Hz; HR: heart rate; LF: low frequency band power of 0.04-0.15 Hz; RR: respiratory rate; RRF: respiratory rate faster than 21 times/min; SDNN: standard deviation of heartbeat interval; TP: total power of heartbeat interval; ULF: ultralow frequency band power of <0.0033 Hz; VLF: very low frequency band power of 0.0033-0.04 Hz.

Additionally, AUC was used to evaluate the effect of the features on the classification performance. As depicted in Figure 5, the AUCs of all 7-day time-scale monitored features were greater than 0.5 and demonstrated statistical significance (*P*<.001). The highest AUC value was observed for RRF_7_ at 0.955. The order of the classification effects was as follows: RRF_7_ > HF_7_ > RR_7_ > LF_7_/TP_7_ > SDNN_7_ > VLF_7_ > ULF_7_/HR_7_. Notably, there were no statistically significant differences between LF_7_ and TP_7_, ULF_7_, and HR_7_.

**Table 4:** Model evaluation index of the single statistical average of features based on 7 days monitoring.

Features (time=7 days)	Accuracy	Sensitivity	Specificity	Youden index	Area under the curve (95% CI)	*P* value
SDNN^a^	0.745	0.465	0.899	0.364	0.768 (0.759-0.777)	<.001
TP^b^	0.792	0.549	0.925	0.474	0.832 (0.825-0.840)	<.001
LF^c^	0.788	0.555	0.917	0.472	0.829 (0.821-0.837)	<.001
HF^d^	0.830	0.701	0.901	0.602	0.890 (0.884-0.896)	<.001
VLF^e^	0.746	0.414	0.929	0.343	0.724 (0.714-0.734)	<.001
ULF^f^	0.708	0.286	0.940	0.226	0.688 (0.677-0.698)	<.001
HR^g^	0.674	0.227	0.920	0.147	0.669 (0.659-0.679)	<.001
RR^h^	0.830	0.651	0.927	0.578	0.869 (0.862-0.876)	<.001
RRF^i^	0.898	0.759	0.974	0.733	0.955 (0.950-0.959)	<.001

^a^SDNN: standard deviation of heartbeat interval.

^b^TP: total spectral power of heartbeat interval.

^c^LF: low frequency band power of 0.04-0.15 Hz.

^d^HF: high frequency band power of 0.15-0.40 Hz.

^e^VLF: very low frequency band power of 0.0033-0.04 Hz.

^f^ULF: ultralow frequency band power of <0.0033 Hz.

^g^HR: heart rate.

^h^RR: respiratory rate.

^i^RRF: respiratory rate faster than 21 times/min.

### Cutoff Values

Considering that the classification performance of HF_7_, RR_7_, and RRF_7_ was superior to that of other features, which reflected the potential values of such features for the diagnosis of COPD, we further analyzed the cutoff values based on the maximum Youden index for HF_7_, RR_7_, and RRF_7_. This serves as a reference for auxiliary diagnosis in patients with COPD. The determined cutoff value for HF_7_ was 1316.30 nU, which corresponded to sensitivity and specificity values of 0.836 and 0.810, respectively. The cutoff value for RR_7_ was 16.3 times/min, accompanied by sensitivity and specificity values of 0.783 and 0.803, respectively. As for RRF_7_, the designated cutoff value was set at 0.1 minute with corresponding sensitivity and specificity values of 0.938 and 0.925, respectively. Samples exceeding these respective cutoff values would be assigned to the case group, while the others would be assigned to the control group (as illustrated in Table 5).

**Table 5:** Cutoff values of statistical average of respiration rate faster than 21 times/min, high frequency band power of 0.15-0.40 Hz in heart rate variability, and respiration rate.

Features (time=7 days)	Cutoff point	Sensitivity	Specificity	Youden index
High frequency band power of 0.15-0.40 Hz	1316.30	0.836	0.810	0.646
Respiratory rate	16.30	0.783	0.803	0.585
Respiratory rate faster than 21 times/min	0.10	0.938	0.925	0.864

## Discussion

### Principal Findings

The primary purpose of this study was to explore the feasibility of bed sensor–aided continuous night-sleep monitoring of vital signs (HRV, HR, and respiration) to assist in the remote diagnosis of COPD. Our findings show that continuous night-sleep monitoring has significant potential in the diagnosis of COPD, and the statistical means of RRF, HF, and RR are the crucial features. By extracting statistical features from the continuously recorded HRV, HR, and respiration over 7, 14, and 30 days, the classification performance of the vital signs for COPD diagnosis was evaluated and compared in different timescales, and the cutoff values of the most important features were obtained as a reference point for in-home diagnosis of COPD. Our prospective cohort experimental analysis verified that the classification performance of the diagnosis of COPD increases directionally proportional to the monitoring duration of vital signs recording. This outcome is expected due to the influence of various factors such as environment, daytime emotions, and activities on the recorded vital signs during a single night sleep [26,27]. Comparatively, by increasing the statistical timescale with regard to feature extraction, the derived statistical features can reflect the status of individual health more accurately [28,29]. Under the tradeoff between the performance of diagnosis and the feasibility of continuous monitoring, it is recommended that a 7-day daily home-based continuous monitoring is sufficient to assist in the diagnosis of COPD.

According to previous research, the resting HR and respiration of patients with COPD are higher than those of healthy individuals. However, there is still a lack of literature support regarding the comparison of the correlation between these indicators and patients with COPD [30]. In this study, we discovered that long-term monitored features such as RR, HF, and RRF were more significant for diagnosing COPD than HR alone. Using either RR or HF as features, the classification performance accuracy already exceeded 80%. Furthermore, when utilizing RRF for classification, the accuracy reached 90%. In comparison, if HR was used for classification purposes only, the accuracy dropped to 67%, with a missed diagnosis rate exceeding 70%.

The resting RR for healthy individuals ranges from 12 to 20 beats per minute [31]. However, our findings showed that RR exceeded 16 beats per minute, which can be viewed as an important feature for the diagnosis of COPD. This finding is expected since patients with COPD are more prone to experiencing rapid breathing [32]. The cutoff value for RRF in this study was determined as 0.1 minute, indicating that any presence of rapid breathing during night sleep significantly increases the probability of a COPD diagnosis. The corresponding sensitivity and specificity values reach up to 94% and 93%, respectively.

Patients with COPD exhibit abnormal autonomic nervous function and reduced HRV [33,34]. Studies have demonstrated a decrease in sympathetic regulation among stable patients with COPD, suggesting a potential shift toward parasympathetic regulation [35]. By contrast, our findings show that patients with COPD exhibit an elevated HRV compared to healthy controls. The possible reasons are 2-fold. On the one hand, the HRV indicators of patients with COPD were increased due to cardiac compensation since all patients with severe cardiovascular diseases were excluded in this study. This phenomenon was also validated by a pioneer study [36]. On the other hand, as pointed out by [37], parasympathetic regulation is more active in patients with acute exacerbation of COPD compared to those with stable COPD. In this study, although the events of acute exacerbation in the case group were excluded before analysis, potential labelling errors of the acute exacerbation cases existed, since the recording of periodic follow-up might have been incomplete due to out-of-hospital visits or recall bias, and small amount of monitoring data during acute exacerbations were included, which could account for the observed increase in HRV among patients with COPD.

LF reflects the regulation of the sympathetic nerve, HF reflects the activity of the parasympathetic nerve, and LF/HF reflects the relative activity of the sympathetic nerve and the parasympathetic nerve, that is, the balance between the sympathetic nerve and the parasympathetic nerve [36]. The results of our study showed that there was no statistically significant differences in the LF/HF characteristics between the case group and the control group, which indicated that in patients with COPD, the LF and HF both increased, thus resulting in the relatively constant LF/HF indices. This is also consistent with previous studies, which demonstrated no significant difference between the LF/HF of patients with COPD and the control groups [37].

### Strengths and Limitations

This study is the first prospective observational study based on continuous vital signs monitoring during night sleep to assist in-home diagnosis of COPD. With the help of noncontact sensors placed under the mattress, we performed continuous night sleep monitoring for individuals with COPD and healthy individuals over weeks and employed the statistical means of the monitored HR, respiration, and HRV at different timescales as features instead of the short-term measurement in a single day. Comparatively, the proposed long-term monitoring could reduce the uncertainty and inaccuracy caused by various interference factors present in short-term monitoring sessions and thereby improve the performance of the auxiliary diagnosis of COPD.

This study has some limitations. The majority of the individuals in the case group were patients with COPD recruited from the hospital. Due to the characteristics of COPD, the proportion of old and lean men in the case group was higher, while individuals in the control group were normal primarily. Statistical comparisons revealed uneven distributions regarding age, sex, and BMI at baseline between the 2 groups, potentially introducing a selection bias. To address this issue, we accounted for these factors during data analysis by adjusting them prior to analysis. Consequently, we observed an improvement in the classification performance while observing minor impacts on the comparison of the classification effects for different features among our patients.

### Conclusions

In this paper, we report the utilization of bed sensors for continuous recording of vital signs, including HR, respiration, and HRV during nighttime, thereby aiming to provide auxiliary diagnosis for COPD. Compared to single-time and single-day measurement, the long-term statistical features derived from continuous vital signs recordings demonstrate superior performance in the diagnosis of COPD. Moreover, the longer the timescale used for constructing the statistical features, the better is the classification performance for COPD diagnosis. Feature importance analysis showed that the statistical features of respiration and HRV contributed significantly more than the HR features in the COPD diagnosis. Specifically, RR, RRF, and HF exhibited the highest contribution to COPD diagnosis and hold important reference for home-assisted diagnosis of COPD. Additionally, this study determines the cutoff values for important features, which can provide a reference range for clinical COPD diagnosis. In principle, this design may also be applicable to other cardiovascular diseases such as heart failure, but the important features for disease diagnosis may be different.

## Appendix

Multimedia Appendix 1 Supplementary data.

## Abbreviations

AUC: area under the receiver operating characteristic curve
COPD: chronic obstructive pulmonary disease
GOLD: Global Initiative for Chronic Obstructive Lung Disease
HF: high frequency band power of 0.15-0.40 Hz
HR: heart rate
HRV: heart rate variability
LF: low frequency band power of 0.04-0.15 Hz
ROC: receiver operating characteristic
RR: respiratory rate
RRF: respiratory rate faster than 21 times/min
SDNN: standard deviation of heartbeat interval
TP: total spectral power of heartbeat interval
ULF: ultralow frequency band power of <0.0033 Hz
VLF: very low frequency band power of 0.0033-0.04 Hz
