# Supplementary material for: Continuous Monitoring of Heart Rate Variability and Respiration for the Remote Diagnosis of Chronic Obstructive Pulmonary Disease: Prospective Observational Study
Source: JMIR Mhealth Uhealth. 2024 Jul 18;12:e56226. doi: 10.2196/56226 (PMC11294786; doi:10.2196/56226)
Supplement: Multimedia Appendix 1 [file mhealth_v12i1e56226_app1.docx]

**Table S1**. Comparison of features based on one day monitoring between two groups

| Features  (t=1) | Total (n=13260) | Control group (n=8652) | Case group (n=4608) | *Z* | *P value* |
| --- | --- | --- | --- | --- | --- |
|  | P_50_(P_25_-P_75_) | P_50_(P_25_-P_75_) | P_50_(P_25_-P_75_) |  |  |
| SDNN_1_ | 73.0(61.0-87.0) | 68.0(59.0-80.0) | 85.0(71.0-104.0) | 43.5 | <.001 |
| TP_1_ | 3357.0(2244.3-5134.0) | 2760.0(1969.3-3906.8) | 5275.0(3487.3--8587.5) | 56.2 | <.001 |
| LF_1_ | 808.0(504.0-1347.0) | 639.5(439.0-978.0) | 1395.0(846.3-2293.3) | 56.4 | <.001 |
| HF_1_ | 1107.0(696.0-1732.0) | 853.0(581.0-1223.0) | 1859.5(1382.0-2529.0) | 67.3 | <.001 |
| LF/HF_1_ | 0.78(0.63-0.96) | 0.78(0.63-0.96) | 0.78(0.63-0.96) | -1.1 | .278 |
| VLF_1_ | 816.6(560.0-1279.0) | 727.6(526.7-1026.3) | 1169.0(674.3-2087.8) | 36.3 | <.001 |
| ULF_1_ | 437.8(224.7-860.2) | 381.7(205.4-696.9) | 606.0(281.0-1260.8) | 23.7 | <.001 |
| HR_1_ | 63.0(59.0-68.0) | 62.0(58.0-66.0) | 65.0(61.0-70.0) | 27.8 | <.001 |
| RR_1_ | 16.0(14.0-17.0) | 15.0(14.0-16.0) | 19.0(16.0-20.0) | 66.4 | <.001 |
| RRF_1_ | 0.0(0.0-14.0) | 0.0(0.0-0.0) | 48.0(7.3-158.0) | 96.4 | <.001 |

**Table S2.** Comparison of statistical average of features based on seven days monitoring between two groups

| Features  (t=7) | Total (n=12396) | Control group (n=8001) | Case group (n=4395) | *Z* | *P value* |
| --- | --- | --- | --- | --- | --- |
|  | P_50_(P_25_-P_75_) | P_50_(P_25_-P_75_) | P_50_(P_25_-P_75_) |  |  |
| SDNN_7_ | 73.2(62.6-85.6) | 68.6(60.0-77.6) | 85.4(73.2-103.0) | 49.4 | <.001 |
| TP_7_ | 3461.8(2312.7-4983.8) | 2759.8(2055.4-3846.4) | 5411.0(3705.2-8503.4) | 61.3 | <.001 |
| LF_7_ | 838.8(522.8-1311.6) | 626.6(463.0-970.6) | 1423.4(940.6-2176.8) | 60.7 | <.001 |
| HF_7_ | 1100.1(725.2-1694.7) | 851.4(625.8-1184.1) | 1850.6(1473.4-2383.6) | 71.9 | <.001 |
| LF/HF_7_ | 0.78(0.66-0.93) | 0.78(0.66-0.92) | 0.79(0.65-0.94) | -0.8 | .414 |
| VLF_7_ | 832.8(593.7-1227.7) | 737.3(564.2-989.2) | 1218.8(736.6-2097.2) | 41.3 | <.001 |
| ULF_7_ | 486.8(315.0-770.0) | 426.1(293.1-612.6) | 692.8(402.6-1177.0) | 34.6 | <.001 |
| HR_7_ | 63.2(59.0-67.0) | 62.0(57.8-65.4) | 65.4(61.6-69.8) | 31.2 | <.001 |
| RR_7_ | 15.8(14.2-17.4) | 15.0(14.0-16.0) | 18.4(16.6-20.0) | 68.1 | <.001 |
| RRF_7_ | 0.0(0.0-17.4) | 0.0(0.0-0.0) | 55.0(11.0-158.2) | 96.1 | <.001 |

**Table** **S3.** Comparison of statistical average of features based on fourteen days monitoring between two groups

| Features  (t=14) | Total (n=11422) | Control group (n=7255) | Case group (n=4167) | *Z* | *P value* |
| --- | --- | --- | --- | --- | --- |
|  | P_50_(P_25_-P_75_) | P_50_(P_25_-P_75_) | P_50_(P_25_-P_75_) |  |  |
| SDNN_14_ | 73.5(63.1-85.6) | 68.8(60.3-77.3) | 85.7(73.8-103.3) | 49.6 | <.001 |
| TP_14_ | 3536.3(2322.7-5010.7) | 2749.8(2071.2-3860.1) | 5458.9(3793.9-8622.6) | 60.7 | <.001 |
| LF_14_ | 862.1(525.9-1330.2) | 618.4(469.7-991.2) | 1423.3(967.3-2184.6) | 60.4 | <.001 |
| HF_14_ | 1122.6(727.1-1712.7) | 830.1(636.1-1174.1) | 1861.8(1512.7-2386.2) | 71.1 | <.001 |
| LF/HF_14_ | 0.79(0.66-0.92) | 0.78(0.67-0.91) | 0.80(0.64-0.93) | -1.1 | .286 |
| VLF_14_ | 847.9(600.1-1248.5) | 743.9(565.8-997.6) | 1260.9(758.3-2103.8) | 41.7 | <.001 |
| ULF_14_ | 515.0(347.2-811.1) | 450.3(326.3-622.6) | 741.8(442.5-1253.3) | 35.8 | <.001 |
| HR_14_ | 63.3(59.3-66.9) | 62.1(57.9-65.3) | 65.5(61.8-69.8) | 31.5 | <.001 |
| RR_14_ | 15.8(14.3-17.4) | 15.0(13.9-16.0) | 18.4(16.6-20.2) | 66.7 | <.001 |
| RRF_14_ | 0.0(0.0-22.2) | 0.0(0.0-0.0) | 58.5(13.6-160.3) | 94.6 | <.001 |

**Table S4.** Comparison of statistical average of features based on thirty days monitoring between two groups

| Features  (t=30) | Total (n=9404) | Control group (n=5698) | Case group (n=3706) | *Z* | *P value* |
| --- | --- | --- | --- | --- | --- |
|  | P_50_(P_25_-P_75_) | P_50_(P_25_-P_75_) | P_50_(P_25_-P_75_) |  |  |
| SDNN_30_ | 74.4(63.6-86.3) | 68.1(60.7-77.0) | 85.8(74.7-103.3) | 47.7 | <.001 |
| TP_30_ | 3659.3(2288.0-5134.3) | 2716.6(2073.9-3913.1) | 5534.5(3823.8-8740.2) | 57.3 | <.001 |
| LF_30_ | 918.3(533.4-1356.6) | 591.0(473.5-993.5) | 1460.3(998.6-2203.1) | 57.7 | <.001 |
| HF_30_ | 1204.2(721.5-1767.0) | 809.5(642.3-1184.3) | 1877.1(1553.2-2393.6) | 67.2 | <.001 |
| LF/HF_30_ | 0.79(0.66-0.91) | 0.78(0.67-0.90) | 0.80(0.64-0.92) | -1.3 | .209 |
| VLF_30_ | 872.7(598.7-1258.3) | 729.0(562.2-1000.6) | 1249.6(783.3-2143.1) | 40.4 | <.001 |
| ULF_30_ | 542.6(369.6-853.3) | 466.9(349.5-635.2) | 798.9(457.9-1385.5) | 34.3 | <.001 |
| HR_30_ | 63.5(59.5-66.9) | 62.3(58.1-65.0) | 65.8(61.8-69.6) | 30.9 | <.001 |
| RR_30_ | 16.0(14.4-17.8) | 15.0(13.9-16.0) | 18.5(16.6-20.1) | 62.3 | <.001 |
| RRF_30_ | 0.0(0.0-31.3) | 0.0(0.0-0.0) | 69.0(17.4-162.8) | 87.4 | <.001 |
